# Supplementary material for: Inhibition of Tityus serrulatus venom hyaluronidase affects venom biodistribution
Source: PLoS Negl Trop Dis. 2019 Apr 19;13(4):e0007048. doi: 10.1371/journal.pntd.0007048 (PMC6493768; doi:10.1371/journal.pntd.0007048)
Supplement: S1 Methods — (DOCX) [file pntd.0007048.s001.docx]

**Supporting information**

**Inhibition of *Tityus serrulatus* Venom Hyaluronidase affects venom biodistribution**

Bárbara Bruna Ribeiro de Oliveira-Mendes^1^•; Sued Eustáquio Mendes Miranda^2^•; Douglas Ferreira Sales-Medina^1^; Bárbara de Freitas Magalhães^3,4^; Yan Kalapothakis^1^; Renan Pedra de Souza^1^; Valbert Nascimento Cardoso^2^; André Luís Branco de Barros^2^; Clara Guerra-Duarte^5^; Evanguedes Kalapothakis^1^; Carolina Campolina Rebello Horta^6^*

^1^Departamento de Biologia Geral, Instituto de Ciências Biológicas, Universidade Federal de Minas Gerais, Belo Horizonte, 31270-901, Minas Gerais, Brazil

^2^Faculdade de Farmácia, Universidade Federal de Minas Gerais, Belo Horizonte, 31270-901, Minas Gerais, Brazil

^3^Department of BioSciences, Rice University, Houston, 77005, Texas, USA

^4^CAPES Foundation, Ministry of Education of Brazil, Brasília, 70359-970, Distrito Federal, Brazil

^5^Fundação Ezequiel Dias, Belo Horizonte, 30510-010, Minas Gerais, Brazil

^6^Mestrado Profissional em Biotecnologia e Gestão da Inovação, Centro Universitário de Sete Lagoas, Sete Lagoas, 35701-242, Minas Gerais, Brazil

•Both authors contributed equally to this work.

*Corresponding author: Carolina Campolina Rebello Horta

Address: Av. Marechal Castelo Branco, 2765 - Santo Antônio, Sete Lagoas, 35701-242, Minas Gerais, Brazil.

Phone: 55 31 21062102

Fax: 55 31 21062101

e-mail: carolina.campolina@unifemm.edu.br; carolinacampolina@yahoo.com.br

**S1 Methods. Electrophoresis and Immunoblotting analysis**

*T. serrulatus* venom (TsV, 15 µg) was submitted to electrophoresis under reducing conditions using a 12% (w/v) SDS-PAGE, as previously described^1^. After separation, proteins were transferred to nitrocellulose membranes and submitted to Western blot analysis^2^. Membranes were blocked using Blocking Buffer (1X TBS with 5% w/v nonfat dry milk) for 1 h at 26ºC and then incubated with rabbit anti-hyaluronidase serum or pre-immune serum (1:5,000) in 1X TBST (TBS Tween-20) with 5% nonfat dry milk at 26°C overnight. After washing with TBS, secondary fluorescence-conjugated anti-rabbit IgG in 1X TBST with 5% nonfat dry milk was allowed to bind at 26°C for 2 h. Following incubation, washing with TBST and drying both membranes, the bands were detected using a fluorescent scanner (GE Healthcare Lifesciences).

**References**

1. Lowry OH, Rosebrough NJ, Farr AL, Randall RJ. Protein measurement with the folin phenol reagent. J Biol Chem. 1951;193(1):265–75.
2. Guimarães E, Machado R, Fonseca MdC, França A, Carvalho C, Araújo e Silva AC, et al. Inositol 1, 4, 5-trisphosphate-dependent nuclear calcium signals regulate angiogenesis and cell motility in triple negative breast cancer. PLoS ONE. 2017;12(4):e0175041.
