# Supplementary figures and images for: Inhibition of Tityus serrulatus venom hyaluronidase affects venom biodistribution
Source: PLoS Negl Trop Dis. 2019 Apr 19;13(4):e0007048. doi: 10.1371/journal.pntd.0007048 (PMC6493768; doi:10.1371/journal.pntd.0007048)

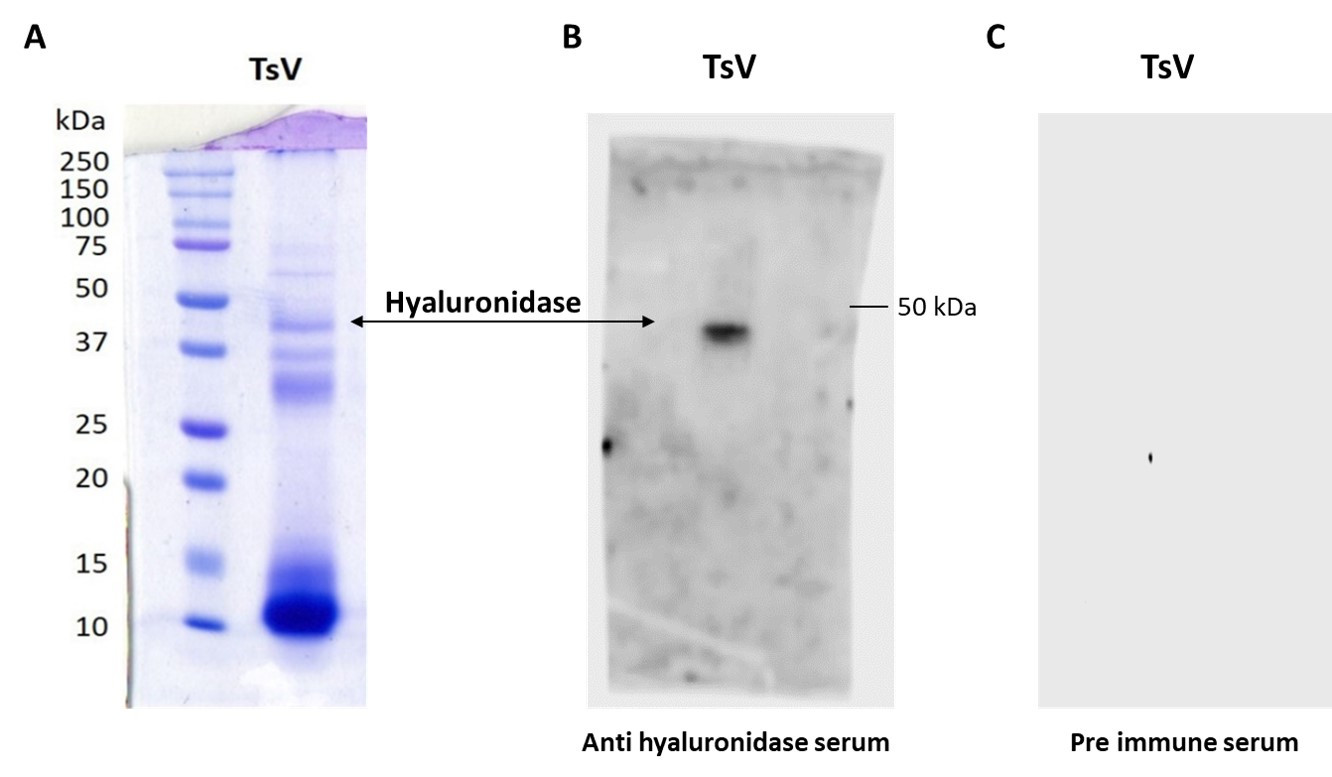

Supplement: S1 Fig — A) 12% (w/v) SDS-PAGE analysis of T. serrulatus venom (15 μg; TsV). B) Immunoblotting of TsV probed with anti-hyaluronidase serum (1:5,000). C) Immunoblotting of TsV probed with pre-immune serum (1:5,000). (TIF) [file pntd.0007048.s002.tif]
